# An improved algorithm to screen for carbapenemase production in *Pseudomonas aeruginosa*

**DOI:** 10.1093/jacamr/dlaf249

**Published:** 2026-01-07

**Authors:** Justin A Ellem, Mitchell J Brown, Indy Sandaradura, Brian P McSharry, Thiru Vanniasinkam

**Affiliations:** Centre for Infectious Diseases and Microbiology Laboratory Services, NSW Health Pathology-ICPMR, Westmead Hospital, Sydney, Australia; School of Dentistry and Medical Sciences, Faculty of Science and Health, Charles Sturt University, Wagga Wagga, Australia; Centre for Infectious Diseases and Microbiology Laboratory Services, NSW Health Pathology-ICPMR, Westmead Hospital, Sydney, Australia; Centre for Infectious Diseases and Microbiology Laboratory Services, NSW Health Pathology-ICPMR, Westmead Hospital, Sydney, Australia; School of Dentistry and Medical Sciences, Faculty of Science and Health, Charles Sturt University, Wagga Wagga, Australia; Gulbali Institute, Charles Sturt University, Wagga Wagga, Australia; School of Dentistry and Medical Sciences, Faculty of Science and Health, Charles Sturt University, Wagga Wagga, Australia

## Abstract

**Objectives:**

One of the biggest challenges for healthcare providers is the difficulty with screening for carbapenemase-producing, carbapenem-resistant *Pseudomonas aeruginosa* (CP-CRPa; *P. aeruginosa*), given the variety of mechanisms that can mediate carbapenem resistance in *P. aeruginosa*. We sought to develop an improved algorithm to screen for carbapenemase activity in *P. aeruginosa* using routine antimicrobial susceptibility testing readily available in most clinical microbiology laboratories.

**Methods:**

Antibiograms of a reference set of *P. aeruginosa* (*n* = 100) with diverse phenotypic and genotypic profiles were compared to determine which antibiotics optimally screen for and differentiate CP-CRPa from CRPa and non-CRPa. The developed algorithm was then applied to 1482 clinical *P. aeruginosa* isolates. Carbapenemase PCR and the modified carbapenem inactivation method were performed on all meropenem-resistant *P. aeruginosa* isolates.

**Results:**

The CP-CRPa screening algorithm developed here uses meropenem, ceftazidime and tobramycin. Carbapenem resistance was identified in 85 (5.7%) isolates, of which 26 (1.8%) were confirmed as CP-CRPa. *bla*_GES_ (57.7%) was the predominant carbapenemase detected, whilst *bla*_NDM_, *bla*_VIM_, *bla*_IMP_ and *bla*_KPC_ carbapenemases were also detected. The CP-CRPa screening algorithm was 100% sensitive (CI_95%_ 84.0%–100%) and 96.6% specific (CI_95%_ 87.3%–99.4%).

**Conclusions:**

We present an antimicrobial susceptibility testing-based screening algorithm that uses meropenem, ceftazidime, and tobramycin to screen for CP-CRPa. When appropriate screening criteria are utilized, confirmatory testing can be significantly reduced, resulting in substantial time and resource savings, without compromising sensitivity, particularly in settings with varying carbapenemase epidemiology.

## Introduction

*Pseudomonas aeruginosa* is a leading cause of healthcare-associated infections, including serious respiratory and bloodstream infections.^1^ A major characteristic of *P. aeruginosa* is its intrinsic resistance to many antibiotics and its ability to acquire new resistance mechanisms.^2^

Carbapenem resistance in *P. aeruginosa* is of significant clinical concern, mediated by several key mechanisms that work individually or in combination to reduce antibiotic efficacy.^3^ Often combined, hyperproduction of chromosomal AmpC cephalosporinases and inactivation or repression of the *OprD* outer membrane porin protein are the major mechanisms causing resistance to essentially all anti-pseudomonal β-lactams, including carbapenems.^4^ A second mechanism is the overexpression of the resistance-nodulation-division efflux pumps, particularly *MexAB-OprM*. The *MexAB-OprM* efflux pump expels most β-lactam antibiotics, including carbapenems, from the cell using a proton gradient to the extracellular environment.^2,5,6^ Acquisition of carbapenemase genes is the third key mechanism, and although the frequency of carbapenemase production in *P. aeruginosa* is still relatively low in many geographical regions, the prevalence of carbapenemase production appears to be increasing.^7^

Screening for carbapenemase-producing, carbapenem-resistant *P. aeruginosa* (CP-CRPa) and distinguishing CP-CRPa from non-CP-CRPa remains challenging, since EUCAST offers no specific guidance for this species.^8^ Consequently, many laboratories either use carbapenem resistance alone as a trigger for screening or omit carbapenemase testing in *P. aeruginosa* entirely. Despite these difficulties, it is important to identify carbapenemases due to the increased transmissibility of CP-CRPa between patients, necessitating the implementation of strict infection control measures.^9^ Additionally, carbapenemase production can have major therapeutic implications, compromising the effectiveness of newly developed β-lactam/β-lactamase inhibitor combinations, resulting in higher rates of mortality.^7^

Given the importance of accurately screening for carbapenemase production, we have developed a practical algorithm using meropenem, ceftazidime and tobramycin to screen *P. aeruginosa* for potential carbapenemase activity using routine antimicrobial susceptibility testing (AST) results by either disc diffusion or determination of minimum inhibitory concentration (MIC) by automated systems such as the BD Phoenix^™^.

## Materials and methods

Antibiograms of 100 reference *P. aeruginosa* isolates (obtained locally unless otherwise cited) with diverse phenotypic and genotypic profiles were compared in this study. These reference strains included: KPC producers (KPC-2 and -3 variants; *n* = 5), GES producers (GES-2 and -5 variants; *n* = 10), IMP producers (IMP-1, -4, -7, -8, -14, -15, -18 and -32 variants; *n* = 10), VIM producers (VIM-1, -2, -3 and -4^10^ variants; *n* = 10), NDM producers (NDM-1, -5 and -7 variants, *n* = 10), AIM-,^11^ SPM-,^12^ DIM-,^13^ FIM-,^14^ SIM- and GIM-producers^14,15^ (*n* = 12; two each). Non-carbapenemase producing strains were used as comparison controls (CRPa: *n* = 23, and non-carbapenem resistant isolates: *n* = 20). Carbapenemase production in these reference isolates was characterized by PCR.

AST was performed on all isolates using the NMIC-422 panel with the BD Phoenix^™^ automated susceptibility system (Becton, Dickinson and Co, Franklin Lakes, NJ, USA). For comparison purposes, disc diffusion was also performed (in accordance with the EUCAST guidelines) with the following antibiotic discs (Oxoid, Hampshire, UK): cefepime (30 μg), ceftazidime (30 μg), ceftolozane-tazobactam (30–10 μg), meropenem (10 μg), piperacillin-tazobactam (30–6 μg) and tobramycin (10 μg). MICs and zone diameters of inhibition (ZDI) were compared for each of the reference isolates to determine the optimal screening cut-off algorithm to screen for carbapenemase production.

Clinical *P. aeruginosa* isolates (*n* = 1482) were collected between 1 August 2023 and 28 February 2024 from hospitals around New South Wales (NSW), Australia, including from hospitals within Western Sydney, Far West NSW, Western NSW, Southern NSW, Hunter New England, and Murrumbidgee Local Health Districts. Isolates were recovered from blood cultures, respiratory, wound and infection control surveillance specimens. Carbapenemase production was phenotypically determined using the modified carbapenemase inactivation method (mCIM) (11), and real-time PCR (*bla*_KPC_, *bla*_SME_, *bla*_GES_, *bla*_IMI_, *bla*_IMP_, *bla*_VIM_, *bla*_NDM_, *bla*_AIM_, *bla*_SPM_, *bla*_SIM_, *bla*_GIM_, *bla*_OXA-23-like_, *bla*_OXA-48-like_, *bla*_OXA-24_ and *bla*_OXA-58_) was performed to determine the carbapenemase genotype of all clinical isolates that were meropenem non-susceptible (MIC ≥ 8 mg/L; ZDI < 14 mm). In this study, carbapenemase production was defined as either a positive mCIM test result or a positive carbapenemase PCR test result. DNA sequencing was used to resolve discrepant results.

## Results

Meropenem non-susceptibility (MIC ≥ 8 mg/L; ZDI < 14 mm) was noted in all 80 reference CP-CRPa and CRPa isolates (Table 1), and in 85/1482 (5.7%) of the clinical *P. aeruginosa* isolates. Ceftolozane-tazobactam susceptibility (MIC ≤ 4 mg/L; ZDI: ≥ 23 mm) was noted in six CP-CRPa reference isolates, whilst 91.3% of non-carbapenemase CRPa reference isolates were also susceptible to ceftolozane-tazobactam. All CP-CRPa reference isolates were non-susceptible to tobramycin with MICs ≥ 8 mg/L (ZDI < 14 mm), whilst no non-CP-CRPa or non-CRPa were non-susceptible with MICs ranging from ≤ 1 to 2 mg/L (ZDI ≥ 18 mm).

**Table 1.** AST resistance distribution of the reference isolates using MICs obtained from the BD Phoenix^™^ and zone diameters of inhibition obtained by disc diffusion

*P. aeruginosa* group	Susceptible % (*n*)						
	FEP	CAZ	C/T	IPM	MEM	TZP	TOB
Non-carbapenem-resistant *P. aeruginosa* (*n* = 20)	75 (15)	75 (15)	100. (20)	100 (20)	100 (20)	85 (17)	100 (20)
Carbapenem-resistant, non-carbapenemase-producing *P. aeruginosa* (*n* = 23)	8.7 (2)	17.4 (4)	91.3 (21)	0 (0)	0 (0)	13 (3)	100 (23)
Carbapenemase-producing, carbapenem-resistant *P. aeruginosa* (*n* = 57)	0 (0)	0 (0)	11 (6)	0 (0)	0 (0)	0 (0)	0 (0)

CAZ, ceftazidime; C/T, ceftolozane-tazobactam; FEP, cefepime; IPM, imipenem; MEM, meropenem; TOB, tobramycin; TZP, piperacillin-tazobactam.

Antibiogram results for the clinical *P. aeruginosa* isolates showed 85/1482 (5.7%) were meropenem non-susceptible. Of these, 28/85 (32.9%) were positive by the carbapenemase screening algorithm, of which 26 were carbapenemase positive by both PCR and mCIM, sensitivity = 100% (CI_95%_ 84.0%–100%) and specificity = 96.6% (CI_95%_ 87.3%–99.4%) (Table 2). The 26 CP-CRPa identified in this study encompassed enzymes of both Class A and Class B carbapenemases, with *bla*_GES_ (15/26, 57.7%) being the most prevalent carbapenemase gene group. Susceptibility to ceftolozane-tazobactam was noted in five clinical CP-CRPa isolates (MICs = 2 mg/L), all of which were identified as *bla*_GES-5_ by DNA sequencing.

**Table 2.** Performance of the different screening algorithms of the 85 carbapenem-non-susceptible *P. aeruginosa* isolates

Algorithm-derived screening criteria (EUCAST)	Number meeting criteria	Carbapenemase producers detected	Carbapenemase producers missed by criteria	Sensitivity (CI_95%_)	Specificity (CI_95%_)	Algorithm reference
MEM (MIC ≥ 8 µg/mL) and CAZ (MIC ≥ 8 µg/mL) and TOB (MIC ≥ 8 µg/mL)	28	26	0	100 (84.0–100)	96.6 (87.3–99.4)	Defined in this study using the reference isolates.
MEM R and CAZ + FEP + C/T NS	32	21	5	80.8 (60.0–92.7)	81.4 (68.7–89.9)	^16^
IPM + MEM R and CAZ + FEP + TZP NS followed by C/T + CZA R^a^	32^a^	21^a^	5^a^	80.8 (60.0–92.7)	81.4 (68.7–89.9)	^17^
IPM or MEM R and CAZ or FEP NS	85	26	0	100 (84.0–100)	0 (0–7.6)	^18^
IPM or MEM R CAZ and/or FEP NS and C/T NS	32	21	5	80.8 (60.0–92.7)	81.4 (68.7–89.9)	^18,19^

CAZ, ceftazidime; C/T, ceftolozane-tazobactam; CZA, ceftazidime-avibactam; FEP, cefepime; IPM, imipenem; MEM, meropenem; NS, non-susceptible; R, resistant; TOB, tobramycin.

^a^CZA not included on the BD Phoenix^™^ NMIC 422 panel.

## Discussion

One of the biggest challenges for healthcare providers is the difficulty with screening for CP-CRPa given the variety of mechanisms that can mediate carbapenem resistance in *P. aeruginosa* and the absence of screening guidelines by EUCAST for *P. aeruginosa*.

Incorporation of ceftolozane-tazobactam non-susceptibility into established screening algorithms using carbapenem resistance plus ceftazidime and/or cefepime and/or piperacillin-tazobactam non-susceptibility was conducted in studies by Gill *et al*.^17^ and Vallabhaneni *et al.*^18^ to increase the sensitivity and specificity of the original screening algorithms, obtaining 100% sensitivity and 89% and 86% specificities, respectively, with the identification of Class B (MBL)-type carbapenemases. Additionally, Mancini *et al.*^19^ showed that ceftolozane-tazobactam alone could effectively screen for Class B carbapenemases in CRPa isolates exhibiting resistance to meropenem and/or imipenem, cefepime and ceftazidime, due to the hydrolysis of ceftolozane *in vitro* without the inhibition by tazobactam.^20^

Although *bla*_VIM_ remains the most frequently identified carbapenemase in *P. aeruginosa*, particularly in South and Central America,^7^ the prevalence of *bla*_GES_ carbapenemases is increasing globally, especially in the Middle East and Africa,^21^ Europe^22^ and Asia.^23^ In our study, both Class A and Class B carbapenemases were identified, with *bla*_GES_ the most common. While ceftolozane-tazobactam could be used as a screening agent for carbapenemases in *P. aeruginosa*, ceftolozane-tazobactam activity against CP-CRPa has been described,^21,24–26^ including with GES-, VIM- and NDM-producing CP-CRPa.^26^ Notably, five *bla*_GES-5_ CP-CRPa were susceptible to ceftolozane-tazobactam in this study.^26^ Gill *et al*.^17^ described a *bla*_GES-20_ CP-CRPa susceptible to ceftolozane-tazobactam, while another study found 13/20 CP-CRPa isolates missed by the ceftolozane-tazobactam non-susceptibility algorithm were GES carbapenemase producers.^27^ Similarly, Ocal *et al.*^28^ reported that 4/33 CIM-positive isolates did not meet the algorithm’s ceftolozane-tazobactam non-susceptibility criteria, while Mancini *et al*.^19^ reported that screening cut-offs of <23 mm failed to identify *bla*_KPC_- and *bla*_GES_ CP-CRPa. Non-susceptibility to ceftolozane-tazobactam has also been reported in *P. aeruginosa* harbouring GES-type carbapenemases,^26^ including a CP-CRPa carrying *bla*_GES-6_.^29^

In contrast, our study identified an alternative algorithm using meropenem, ceftazidime and tobramycin (MICs ≥ 8 mg/L; disc diffusion zone diameters: meropenem and tobramycin <14 mm, and ceftazidime < 17 mm) that reliably screen for CP-CRPa carrying either Class A or Class B carbapenemases. Tobramycin resistance in multi-drug-resistant *P. aeruginosa* has been described previously,^30^ typically mediated by aminoglycoside 6′*N*-acetyltransferases (AAC(6′)) alone or in combination with aminoglycoside adenylyltransferases.^31^ These enzymes, combined with carbapenemase resistance genes, have been widely reported.^23,32^ To our knowledge, however, the use of meropenem, ceftazidime and tobramycin in combination as screening agents for CP-CRPa, has not been previously described.

Although carbapenems alone lack specificity in distinguishing CP-CRPa from non-CP-CRPa, as shown in this and other studies,^17,18,28^ their inclusion at optimal concentrations remains a critical component of any effective screening algorithm.^33^

Vega *et al.*^16^ proposed including ceftazidime-avibactam into screening algorithms to increase the specificity of carbapenemase screening. Although ceftazidime-avibactam was not included in this study since it is not included on the BD Phoenix^™^ NMIC-422 panel, we agree with Gill *et al*.^17^ that the inclusion of ceftazidime-avibactam will likely decrease sensitivity in settings where carbapenemases other than Class B carbapenemases are prevalent. Consequently, the inclusion of ceftazidime-avibactam should only be used in combination with other screening criteria to distinguish Class B carbapenemases from Class A and D carbapenemases.^17^

The algorithm outlined in this study does not require additional or specialized antimicrobial susceptibility testing and can be used regardless of the AST methodology employed locally in clinical microbiology laboratories with the provision of both MIC and zone diameter of inhibition screening cut-off criteria. Furthermore, this algorithm can be used in settings with varying carbapenemase epidemiology. By significantly reducing the number of isolates requiring confirmatory carbapenemase testing, this algorithm facilitates the provision of results sooner at a reduced cost. Consequently, appropriate treatment options can be initiated earlier, and infection prevention and control measures implemented sooner.

The main limitation of this study is the low prevalence of CP-CRPa isolates in our local setting, and the predominance of GES-type carbapenemases. Although the prevalence of GES-type carbapenemases in *P. aeruginosa* is increasing, particularly in Asia,^23^ the Middle East and Africa,^21^  *bla*_VIM-2_ is the most common carbapenemase found in *P. aeruginosa* in Europe, USA and South and Central America.^7^ Integrons capture gene cassettes encoding antimicrobial resistance and have emerged as critical elements in the dissemination of antimicrobial resistance within *P. aeruginosa.*^34^  *bla*_VIM-2_ has frequently been reported within gene cassette arrays of Class 1 integrons, with various aminoglycoside modifying enzymes, particularly *aac* and*/or aad* genes, situated at the 5′ and/or 3′ of the carbapenemase resistance gene.^35–39^ As such, future studies should be undertaken on a larger cohort to develop further understanding of the epidemiology across various geographical regions, to test the strength of the algorithm described here.

A further limitation of this study is the unknown impact on the algorithm described in this study, when carbapenemase genes and aminoglycoside modifying enzymes are carried on separate mobile genetic elements, providing for independent acquisition and transmission. A final limitation to this study is that broth microdilution was not performed to confirm the BD Phoenix^™^ and disc diffusion AST results, on the basis that both the BD Phoenix^™^ and disc diffusion methods are standardized, guideline-validated methods with established accuracy and reproducibility.
